# Hypoxic Stress Induced by Hydralazine Leads to a Loss of Blood-Brain Barrier Integrity and an Increase in Efflux Transporter Activity

**DOI:** 10.1371/journal.pone.0158010

**Published:** 2016-06-23

**Authors:** Morgane Chatard, Clémentine Puech, Frederic Roche, Nathalie Perek

**Affiliations:** 1 Université de Lyon, UJM-Saint-Etienne, SNA-EPIS, EA4607, F-42023, Saint-Etienne, France; 2 Université de Lyon, UJM-Saint-Etienne, INSERM, SAINBIOSE U1089 Team DVH, F-42023, Saint-Etienne, France; Hungarian Academy of Sciences, HUNGARY

## Abstract

Understanding cellular and molecular mechanisms induced by hypoxic stress is crucial to reduce blood-brain barrier (BBB) disruption in some neurological diseases. Since the brain is a complex organ, it makes the interpretation of *in vivo* data difficult, so BBB studies are often investigated using *in vitro* models. However, the investigation of hypoxia in cellular pathways is complex with physical hypoxia because HIF-1α (factor induced by hypoxia) has a short half-life. We had set up an innovative and original method of induction of hypoxic stress by hydralazine that was more reproducible, which allowed us to study its impact on an *in vitro* BBB model. Our results showed that hydralazine, a mimetic agent of the hypoxia pathway, had the same effect as physical hypoxia, with few cytotoxicity effects on our cells. Hypoxic stress led to an increase of BBB permeability which corresponded to an opening of our BBB model. Study of tight junction proteins revealed that this hypoxic stress decreased ZO-1 but not occludin expression. In contrast, cells established a defence mechanism by increasing expression and activity of their efflux transporters (Pgp and MRP-1). This induction method of hypoxic stress by hydralazine is simple, reproducible, controllable and suitable to understand the cellular and molecular mechanisms involved by hypoxia on the BBB.

## Introduction

The blood-brain barrier (BBB) is a multicellular neurovascular unit formed of brain microvascular endothelial cells which are surrounded and supported by astrocytes, pericytes and extracellular matrix[1]. Brain microvascular endothelial cells control the transport of substances between blood and the brain via efflux pumps (transcellular transport) and tight junction (TJ) complexes (paracellular transport)[2]. This specialized phenotype allows a suitable protection for the brain[3–5]. Moreover, these barrier properties are mostly induced and maintained by the close opposition between brain microvascular endothelial cells and astrocytes[6,7]. During the last decades it was described that BBB disruption contributed to the pathophysiology of some neurological diseases such as Alzheimer’s disease, multiple sclerosis, Parkinson’s disease and stroke. Since the brain is a complex organ, it makes the interpretation of *in vivo* data difficult, so BBB studies are often investigated using *in vitro* models[8,9]. Hypoxia is a common feature that characterizes many of these diseases and represents a major stress factor that leads to BBB disruption[2,10,11]. The cellular response to hypoxia is mainly driven through the activation of the hypoxia-induced factor 1 (HIF-1) pathway[12,13]. Under normoxic conditions, oxygen regulates the HIF-1α subunit which is rapidly degraded by prolyl hydroxylation that targets its degradation in the proteasome. Hypoxia inhibits the prolyl hydroxylase domain leading to stabilization of the HIF-1α subunit in the cytoplasm. Then it is translocated to the nucleus where it binds to hypoxia responsive elements in promoter regions of target genes involved in cellular adaptation to hypoxic stress and induces their expression[12]. HIF-1α seemed to be a key factor to decrease the BBB’s permeability[13]. Elucidation of the cellular and molecular mechanisms induced by hypoxic stress is complex with physical hypoxia because HIF-1α has a short half-life. In this regard, a wide variety of prolyl hydroxylase domain (PHD) inhibitors, which lead to a stabilization of HIF-1α, have been developed. These inhibitors allow to create hypoxic stress and represent a useful method to investigate the BBB’s disruption by hypoxia. The most used in the literature is cobalt chloride (CoCl_2_)[12,14,15]. Cervelatti et al, used CoCl_2_ to achieve stabilization of HIF-1α because it inhibits PHD by blocking the catalysis of prolyl hydroxylases[16]. However, CoCl_2_ is a rather highly cytotoxic agent for some cell types because CoCl_2_ activates caspase-3 which leads to apoptosis[16]. Hydralazine is a vasodilator used to treat severe hypertension, congestive heart failure, myocardial infarction and preeclampsia[17]. Hydralazine also shows a capacity to induce a transient and physiological HIF-1α overexpression by inhibiting PHD activity[18]. In the literature, hydralazine was only used to mimic a hypoxic state in *in vivo* and *in vitro* cancer models[19]. Hydralazine could represent a suitable and innovative way to study the cellular mechanism involved in hypoxic stress on the BBB and thereby understand the BBB disruption observed in several neurological diseases.

In the present study, we evaluated and validated the potentiality of hydralazine as a hypoxia mimetic agent in comparison to physical hypoxia (standard method of hypoxia induction). Impact of hypoxic stress induced by hydralazine and physical hypoxia on BBB integrity was determined using a coculture in-contact model composed of the immortalized cell line bEnd.3[20] and the C6 cell line (rat malignant glioma cells which display astrocytic properties[9]). This approach allowed interaction between endothelial cells and astrocytic cells. Then impact of hypoxic stress was assessed by studying endothelial paracellular permeability with transendothelial electrical resistance (TEER) measurements and absolute membrane permeability was determined with sodium fluorescein (Na-F)[21,22]. Evaluation of transport was also investigated on expression and activity of two efflux transporters (Pgp and MRP-1) and two TJ proteins (ZO-1 and occludin). Our results showed that hydralazine represented a suitable, original and reproducible way to create a reproducible hypoxic environment since it had the same effect as physical hypoxia. This mimetic agent was further used to evaluate the impact of hypoxic stress on the integrity of our BBB model to understand the cellular mechanism involved by hypoxia.

## Materials and Methods

### Chemicals and reagents

Hydralazine, BCECF-AM, probenecid, verapamil and Na-F were purchased from Sigma-Aldrich (St Quentin Fallavier, France). MTT (methyl-thiazolyl-tetrazolium) kit and DMEM (Dulbecco’s Modified Eagle’s Medium) were also purchased from Sigma. All reagents for the LDH release test were purchased from Promega Corporation (Madison, USA). Antibodies and reagents for the detection of HIF-1α were products of R&D systems (Lille, France). All compounds for Ringer-Hepes buffer were purchased from Sigma.

Occludin and ZO-1 antibodies were purchased from Life Technologies (Saint Aubin, France), Pgp and MRP-1 antibodies were purchased from GeneTex (San Antonio, Texas, U.S.A) and Santa Cruz Biotechnology (Dallas, Texas, U.S.A), respectively.

bEnd.3 cells and C6 cells were obtained from the ATCC (Manassas, VA, USA).

Cell culture inserts for 24-well (0.4 μm pore diameter size, transparent PET membrane) were purchased from Corning distributors (Sigma). EVOM voltohmmeter system was purchased from World Precision Instruments (Hertfondsire, UK).

### *In vitro* cytotoxicity assay

Cytotoxicity of hydralazine was measured by MTT method to determine the least cytotoxic concentration. Growing cells were seeded at 10,000 cells/well using a 96-well microplate that was supplemented with 100 μl DMEM. Cells were allowed to grow for 48 h before they were exposed to drugs. Then cells were treated with various concentrations of hydralazine. Phosphate buffer saline (PBS) 1X was used to dissolve drugs. After the treated cells were incubated for 24 h, 20 μl MTT was added and the plates were incubated at 37°C for 4 h. To dissolve formazan, 100 μl DMSO was added and the plates were measured at 570 nm with a spectrophotometer. The least cytotoxic values were determined by plotting the drug concentrations versus the survival ratio of treated cells.

Then we also evaluated cell death with the LDH release method to confirm the least cytotoxic concentration of hydralazine given by MTT results. For that, cells were seeded in 96-well plates at 10,000 cells/well. 100 μM of hydralazine was added during 2h (time of stress hypoxic that was used in this study) and 24 h (time chosen to determine the least cytotoxic concentration). After exposure period, a lysis solution was used in control wells to generate a maximum LDH release. Then cells were incubated with cytotox-ONE^™^ reagent during 10 min. A stop solution was added before recording fluorescence with an excitation wavelength of 560 nm and an emission wavelength of 590 nm. The average fluorescence values of the culture medium background (wells without cells) were substracted from fluorescence values of experimental wells. Then percent cytotoxicity was given by the following equation:

$$\%cytotoxicity=100x\frac{\left(\text{experimental}-\text{culture medium background}\right)}{\text{maximum LDH release }-\text{ culture medium background)}}$$

### Exposure to physical hypoxia

To induce hypoxia, our *in vitro* BBB model was placed into a special chamber equipped with a thermostat housing that allowed us to incubate our cells at 37°C. Then cells were exposed to either normoxic or hypoxic conditions (2% O_2_), as was previously described by Fischer et al[9].

### Effect of hydralazine treatment or physical hypoxia on hypoxia metabolic pathway

Effect of hydralazine treatment or physical hypoxia was validated by the study of HIF-1α expression (the key regulation of hypoxic response) to determine the effective dose where HIF-1α was induced. For that, cells were seeded onto a 96-well plate at a density of 10,000 cells/well. Then cells were treated with hydralazine (50 and 100μM, the least cytotoxic doses defined by cytotoxic test) or exposed to physical hypoxia during 2 h. Finally, cells were fixed to the support with 4% formaldehyde and the expression of HIF-1α was determined by whole cell-ELISA. For that, anti-HIF-1α antibodies were added at 4°C overnight (diluted at 1/100), then cells were incubated with secondary antibodies for 2 h at room temperature (diluted at 1/500). Fluorescence was measured with a fluorescence spectrophotometer with 540 nm excitation and 600 nm emission wavelengths.

Furthermore, hydralazine is a vasodilator used for treating severe hypertension. So we verified the specificity of hydralazine to produce a cellular hypoxic state by comparing it to another vasodilator, prazosin, as a negative control.

### *In vitro* BBB model set up

The BBB model was composed of bEnd.3 cells (immortalized mouse brain endothelial cells) and C6 cells. All cells were used before passage number 30 (passages 12–28 for bEnd.3 cells and 10–30 for C6 cells), which corresponded to the time where these cells may begin to lose their BBB properties, as cited by the supplier and in a previous study [23].

**Fig 1:**
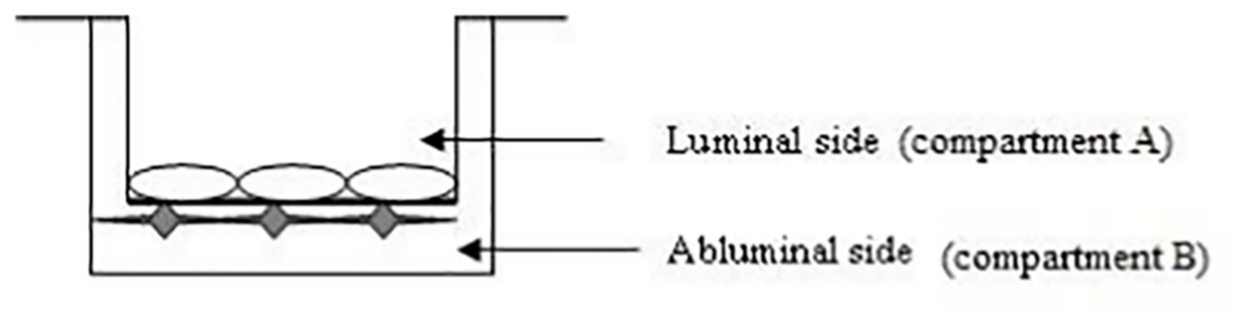
Schema of the *in vitro* BBB model.

Once the cells reached subconfluency, they were placed onto cell culture inserts for 24-well plates. Fig 1 shows a schematic description of the *in vitro* BBB model.

For the procedure of contact coculture, the transwell filter was inverted and C6 cells were seeded onto the abluminal side of the filter at a density of 4x10^4^ cells/cm^2^. Then cells were placed at 37°C for 6 h (time necessary for the cells to adhere to the membrane of the insert). Afterwards, the insert was flipped back and C6 cells were cultured for two days in DMEM. At the end of two days, bEnd.3 cells were seeded onto the luminal side of the transwell filter at a density of 4x10^5^ cells/cm^2^ and cocultured with C6 cells for eight days.

### TEER measurements

To characterize the formation of a tight endothelial cell monolayer, TEER was recorded using an EVOM resistance meter. One electrode was placed on the luminal side and the other electrode on the abluminal side. These two electrodes were separated by the endothelial layer. The TEER measurements of blank filters (without cells) were subtracted from those of filters with cells. Then the resulting value was multiplied by the membrane area to obtain the TEER measurements in Ω.cm^2^.

### Sodium fluorescein permeability measurements

Endothelial paracellular barrier function was also evaluated by measuring the permeability of cells to Na-F (MW = 376 Da). First the medium was removed and cells were washed with Ringer-Hepes prewarmed buffer (5 mM Hepes, 5.2 mM KCl, 2.2 mM CaCl_2_, 0.2 mM MgCl_2_, 6 mM NaHCO_3_ and 2.8 mM glucose). Then Ringer-Hepes buffer containing 10μg/ml of Na-F was loaded onto the luminal side of the insert and incubated at 37°C for 1 h. Samples were removed from the abluminal chamber at 10, 20, 30, 40, 50 and 60 min and immediately replaced with fresh Ringer-Hepes buffer. The concentration of Na-F was determined using a fluorescence multiwell plate reader (Ex (λ) 485 nm; Em (λ) 530 nm). Transendothelial permeability coefficient (Pe) was calculated as previously described by Deli et al[24]. The increment in cleared volume was calculated by dividing the amount of compound in the receiver compartment by the drug concentration in the donor compartment. The volume cleared was plotted versus time and the slope estimated by linear regression analysis. Then the average cleared volume was plotted versus time, and permeability x surface area product value for endothelial monolayer (PSe) was calculated as follows:

$$\frac{1}{PSendothelial}=\frac{1}{PStotal}-\frac{1}{PSinsert}$$

Where PS total corresponded to the experimental data and PS insert corresponded to the insert without endothelial cells (insert with only C6).

PS_endothelial_ divided by the surface area (A) in cm^2^ (0.33cm^2^ for a 24-transwell) generated the endothelial permeability coefficient (Pe in 10^−6^ cm.s^-1^) and calculated as follows:

$$Pe=\frac{PSendothelial}{A}$$

### Whole cell ELISA of Pgp, MRP1, ZO-1 and occludin

Cocultured cells were washed with 1% BSA in PBS at pH 7.4 and fixed for 20 min at room temperature with 4% paraformaldehyde in PBS at pH 7.4. Then inserts were washed again and overlaid with 3% H_2_O_2_ in methanol for 30 min to block endogenous peroxidase, followed by 20% normal goat serum to block unspecific immunoglobulin binding. Cells were incubated all night at 4°C with either monoclonal mouse anti-Pgp (2 μg/ml), monoclonal mouse anti-MRP-1 (10 μg/ml), rabbit anti-ZO-1 (4 μg/ml) or rabbit anti-occludin (1 μg/ml) antibodies. Then cells were washed and peroxidase conjugated anti-mouse IgG was added as a secondary antibody for 2 h at room temperature (diluted at 1/500). After washing several times, 0.1% of o-phenylenadiamine and 0.002% H_2_O_2_ in 0.05 M citrate buffer at pH 4.5 was added for 10 min at room temperature and in the dark. The colour reaction was measured with a spectrophotometer at 490 nm, as described by Cioni et al [3].

### Drug transporter functional assays

The functionality of Pgp and MRP-1 was tested by assessing the release of the substrate BCECF-AM (specific for Pgp and MRP-1) in the absence or presence of specific inhibitors such as verapamil for Pgp and probenecid for MRP-1. The cocultured cells were washed and cultured in DMEM. Then cells were preincubated for 15 min in the absence or presence of inhibitors at 37°C (40 μM verapamil and 1 mM probenecid, concentrations were determined previously[25]). Inhibitors were added in the luminal compartment to study the transport from the luminal to the abluminal side and conversely. The inserts were incubated with 1 mM of BCECF-AM for 1 h at 37°C. Finally, 100 μl was sampled from each compartment and fluorescence was measured with a fluorescence spectrophotometer at 493 nm excitation and 515 nm emission wavelengths.

### Statistical analysis

The values are expressed as the means ± s.e.m. Statistical analysis was performed using Student’s t-test. One-way and two-way analyses of variance (ANOVA) followed by Tukey-Kramer’s tests were applied to multiple comparisons. Statistical analysis was performed using Statview software. The differences between means were considered to be significant when P values were less than 0.05.

## Results

### *In vitro* cytotoxicity assay

**Fig 2:**
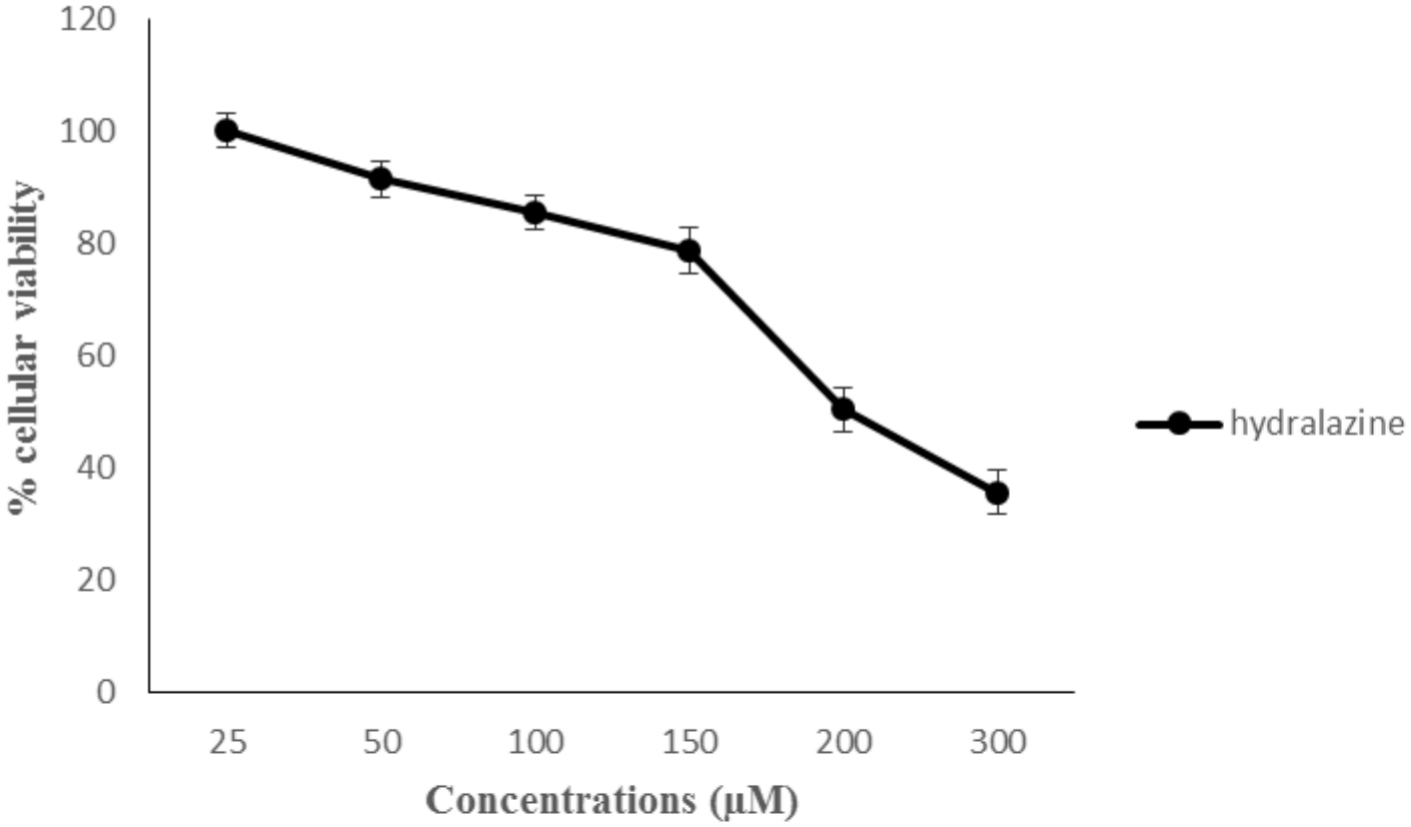
Cytotoxicity effect of drugs in bEnd.3 cells. Cells were incubated with various concentrations of hydralazine for 24 h. Cytotoxicity was measured by an MTT assay. The results are presented as mean value for triplicate.

The incubation of 24 h allowed us to determine the least cytotoxic concentration that we should use in our study. bEnd.3 cells were exposed to various concentrations of hydralazine (25–300 μM). Cytotoxicity activity was evaluated, in a first time, by the MTT method (Fig 2). IC_50_ value of hydralazine was 200 μM. We chose to use 100 μM of hydralazine for our study because at this concentration we had few cytotoxicity effects and confirmed Knowles’s results [18]. Then we could confirm that 100 μM of hydralazine was least cytotoxic by evaluating cell death with the LDH release method (S1 Fig).

### Effective dose of drugs induced HIF-1 pathway

**Fig 3:**
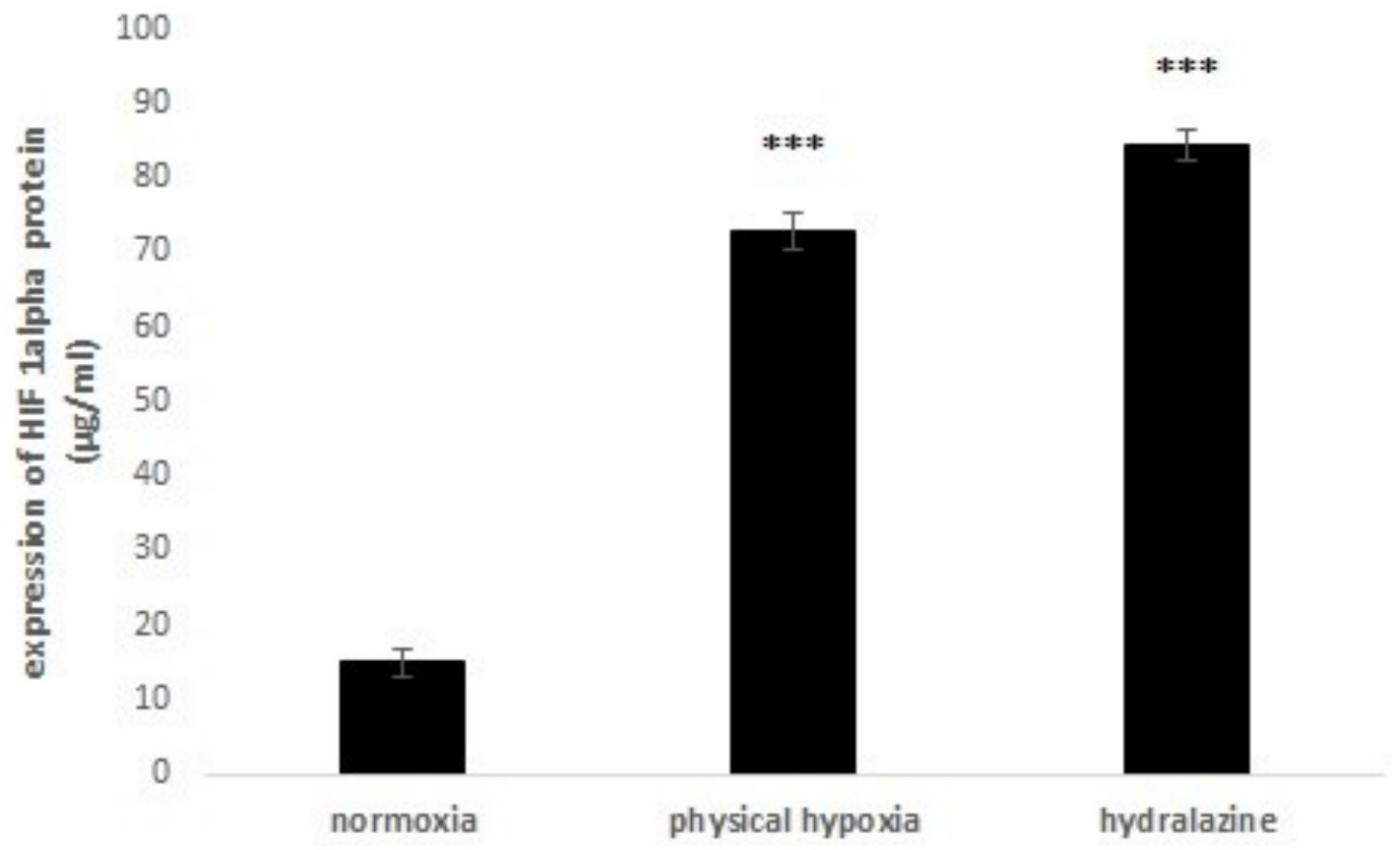
HIF-1 expression induced by each exposure. bEnd.3 cells were exposed to 100 μM hydralazine or physical hypoxia. Results were presented as mean value ± s.e.m (n = 8) and normalized. *** P < 0.001 normoxia vs. hypoxia.

Hydralazine is a chemical agent which could mimic hypoxia since it inhibited PHD that negatively regulates HIF-1. First we determined the effective dose of hydralazine which induced HIF-1α overexpression. For that, bEnd.3 cells were exposed to 50 and 100 μM of hydralazine during 2 h and we compared it to the expression of HIF-1α after exposure to 2% 0_2_. The concentration 50 μM of hydralazine did not induce HIF-1α (data not shown). After 2 h treatment of 100 μM hydralazine, the level of HIF-1α significantly increased to 84.5 ± 2.04 μg/ml (versus 15 ± 1.8 μg/ml for normoxia) (p < 0.001); whereas physical hypoxia significantly induced HIF-1α after 2 h of exposure with an increase to 72.86 ± 2.6 μg/ml (versus 15 ± 1.8 μg/ml for normoxia) (p < 0.001) (Fig 3). Consequently, we used the concentration of 100 μM and 2 h of exposure in our study.

To determine the specific action of the vasodilator hydralazine as a hypoxia mimetic, we compared hydralazine with another vasodilator, prazosin (data not shown). The results showed that the addition of prazosin had no effect on the expression of HIF-1α and, therefore, did not have an effect on the hypoxia metabolic pathway.

### Impact of physical hypoxia and hydralazine on BBB integrity

#### TEER measurements

**Fig 4:**
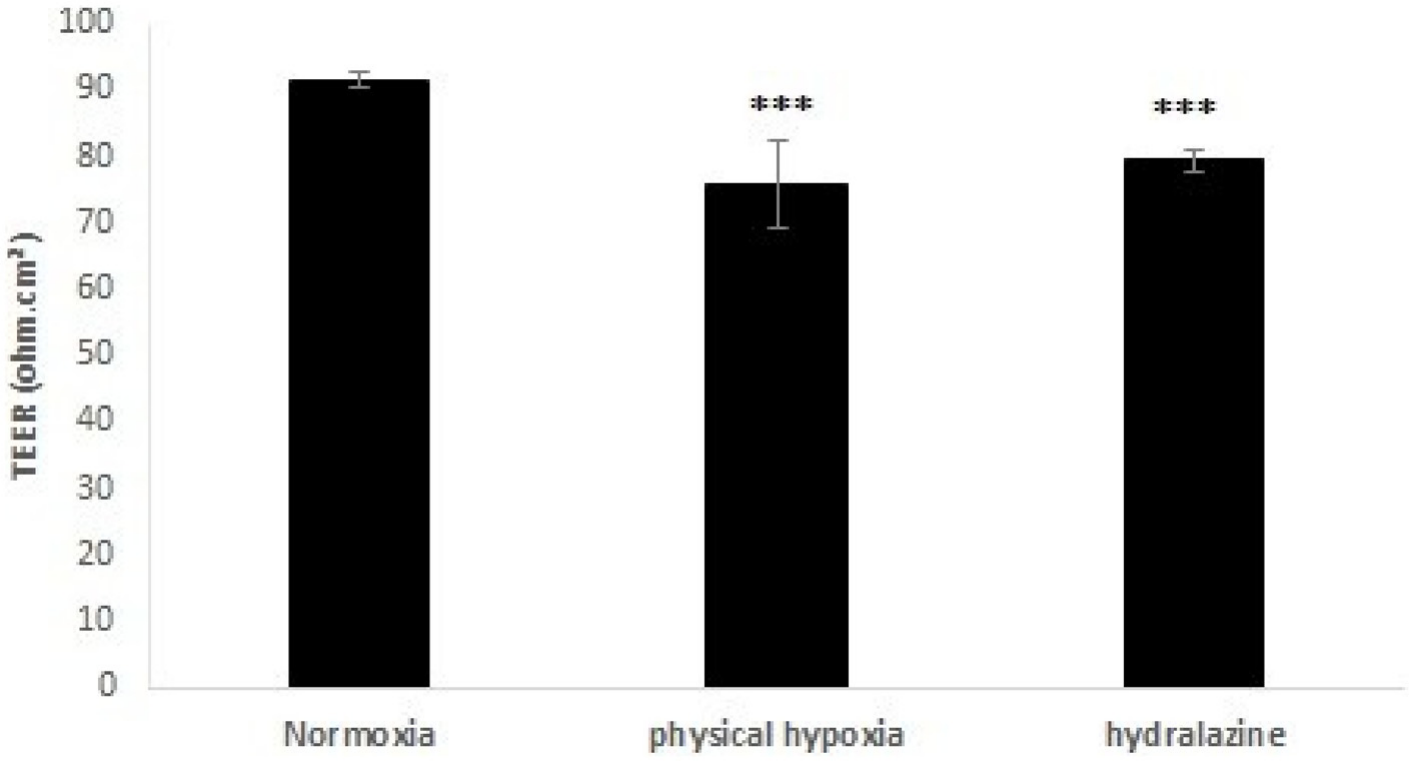
Transendothelial electrical resistance (TEER) measurement after cells of the BBB model were exposed to 100 μM hydralazine or physical hypoxia during 2 h. Results were presented as mean value ± s.e.m (n = 6). *** P < 0.05 normoxia vs. hypoxia.

At the optimal TEER measurement (day 6 established at the laboratory), cells were exposed 2 h to physical hypoxia or hydralazine. Fig 4 shows the impact of these exposures on TEER in an *in vitro* BBB model. A decrease in TEER was observed after 2 h for each exposure. For hydralazine, TEER significantly decreased by 13.3%. TEER values varied from 91.5 ± 1.1 to 79.36 ± 1.5 Ω.cm² (p < 0.001). For physical hypoxia, TEER significantly decreased by 17.2%. TEER values varied from 91.5 ± 1.1 to 75.80 ± 6.4 Ω.cm² (p < 0.001).

#### Membrane permeability

**Fig 5:**
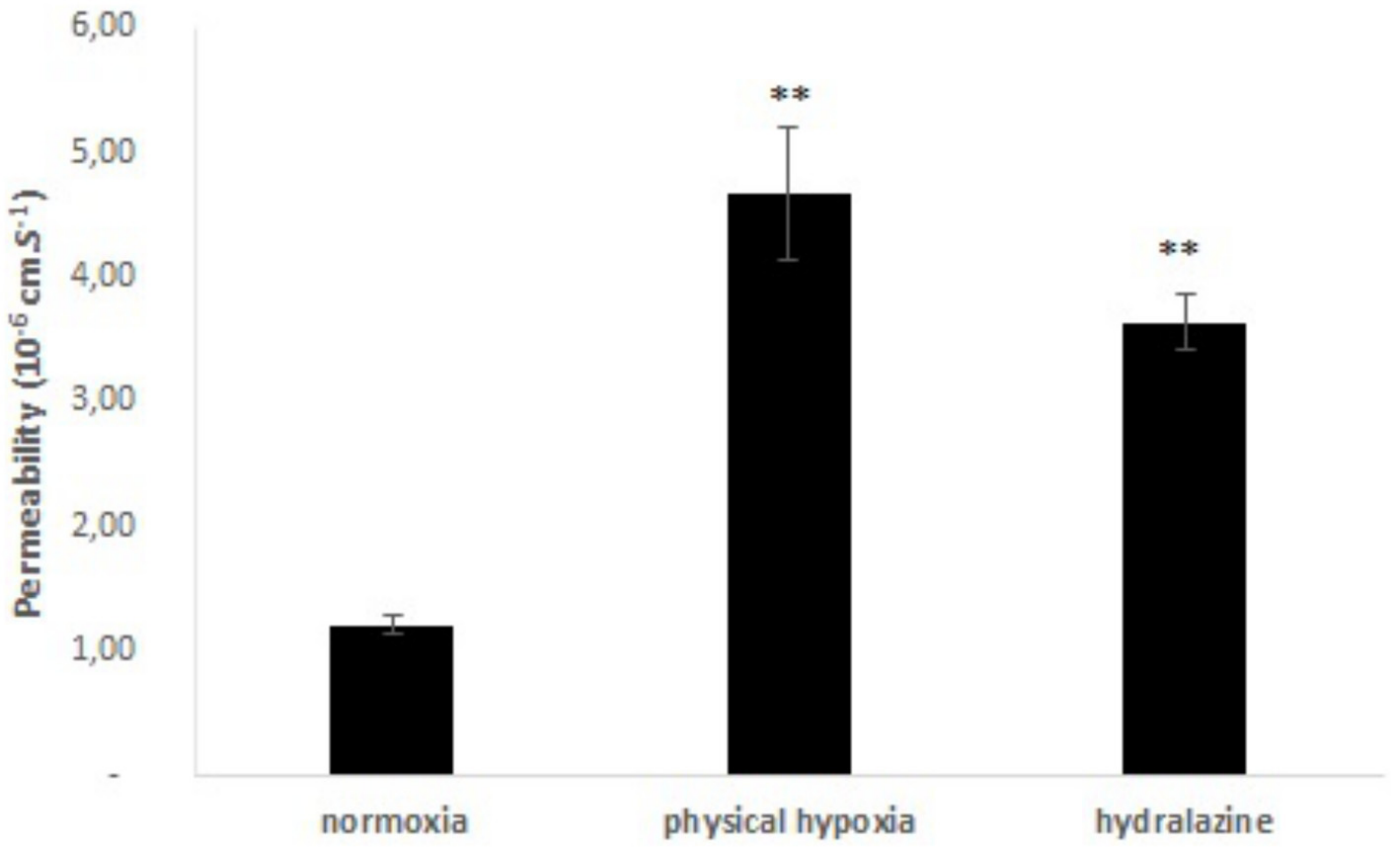
Absolute membrane permeability measurement during 2 h of hydralazine or physical hypoxia exposures versus normoxia. Results were presented as mean value ± s.e.m (n = 4). ** P < 0.01 normoxia vs. hypoxia.

Absolute membrane permeability was determined after 2 h exposure to hydralazine or physical hypoxia, and results are shown in Fig 5. Coefficient permeability of Na-F in the BBB model significantly increased from 1.21 ± 0.08x10^-6^ to 4.67 ± 0.53x10^-6^ cm.s^-1^, for physical hypoxia (p < 0.01). Cells of the BBB model treated with hydralazine showed a significant increase in Na-F permeability (p < 0.01). The permeability coefficient of Na-F varied from 1.21 ± 0.08x10^-6^ to 3.64 ± 0.23x10^-6^ cm.s^-1^. There was no significant difference between the two exposures.

#### Paracellular transport mediated tight junction proteins occludin and ZO-1

**Fig 6:**
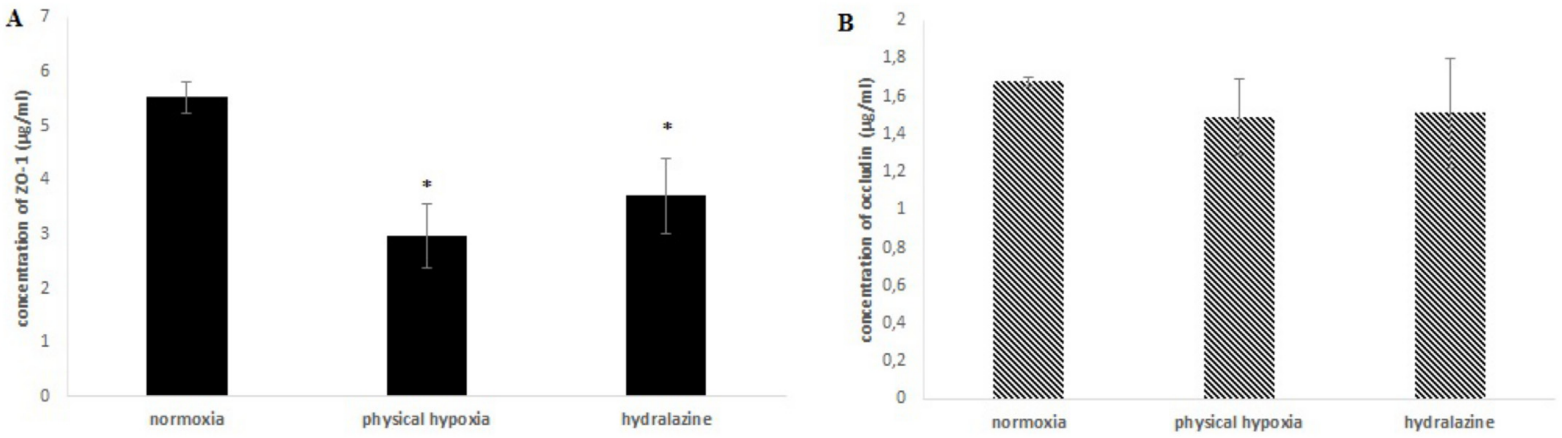
Concentrations of ZO-1 (A) and occludin (B) after cells of the BBB model were exposed to 100 μM hydralazine, or physical hypoxia, during 2h. Results are presented as mean value ± s.e.m (n = 3) and corrected by number of viable cells. * P < 0.05 normoxia vs. hypoxia.

Determination of occludin and ZO-1 expressions in our *in vitro* BBB model were established after hydralazine or physical hypoxia exposures (2h) (Fig 6). Expression of ZO-1 significantly decreased after hydralazine or physical hypoxia exposures (p = 0.028 and p = 0.024, respectively). Expression of ZO-1 decreased by 35% with hydralazine and by 44% with physical hypoxia. There was no significant difference between hydralazine or physical hypoxia. There was also no significant difference for occludin expression with these two exposures. The concentration of occludin varied from 1.68 ± 0.02 to 1.51 ± 0.29 μg/ml with hydralazine and from 1.68 ± 0.02 to 1.49 ± 0.2 μg/ml with physical hypoxia.

#### Transendothelial transport mediated efflux transporters

**Fig 7:**
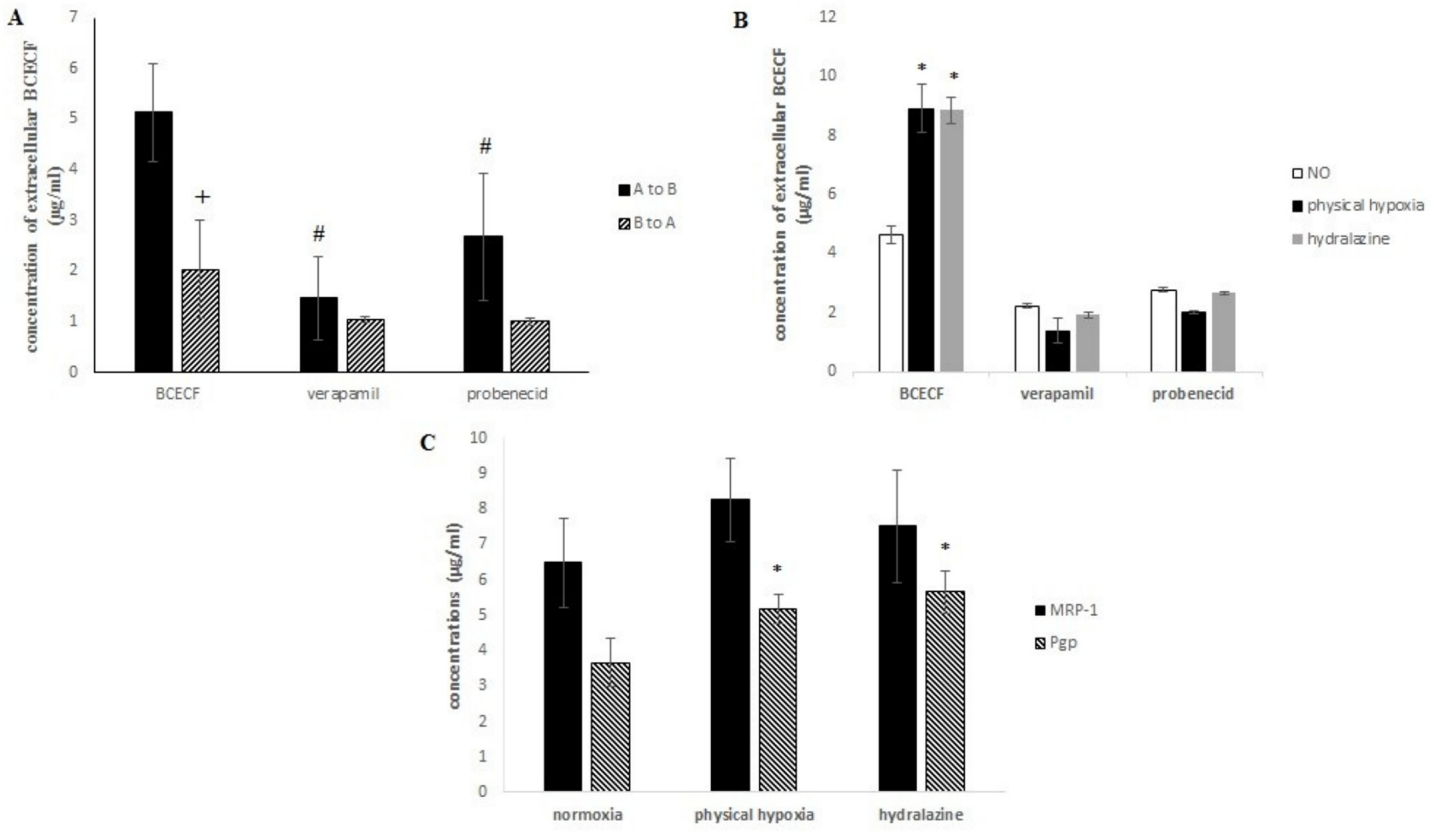
**(A) Transport of BCECF by Pgp and MRP-1 from compartment A to B or B to A. (B)** Release of BCECF during transport from A to B after hydralazine or physical hypoxia exposure. (C) Concentrations of MRP-1 and Pgp after cells of the BBB model were exposed to 100 μM hydralazine, or 2% 0_2_ during 2 h. Results are presented as mean value ± s.e.m (n = 3). ^#^ P < 0.05 BCECF vs. inhibitors and* P < 0.05 normoxia vs. treatment.

Transendothelial transport was evaluated by studying the transport of a substrate of Pgp and MRP-1, *i*.*e*., BCECF-AM, with a method developed in our laboratory[26]. BCECF-AM is cleaved by intracellular esterase into a fluorescent component BCECF. We studied the release of BCECF in the upper compartment A (transport A to B) and in the lower compartment B (transport B to A). Fig 7A shows a significant decrease of extracellular BCECF release during the transport from B to A (p = 0.039). Pumps were more active on apical membranes of bEnd.3 cells (transport from A to B) than on C6’s membranes, since BCECF was more efflux. We also noticed that this release was due to Pgp and MRP-1, since a significant decrease of extracellular BCECF was observed when cells were respectively incubated with verapamil (p = 0.022) or probenecid (p = 0.035), two inhibitors of these pumps. Then transendothelial transport after hypoxic stress was evaluated. We investigated if the transport of BCECF from A to B was changed after hydralazine or physical hypoxia exposure (2h) (Fig 7B). We noticed that efflux transporters prevented the entry of BCECF in cells, since an important transendothelial transport was observed after each exposure. A significant increase of extracellular BCECF was also observed after hydralazine exposure: 4.64 ± 0.29 μg/ml versus 8.84 ± 0.47 μg/ml (p = 0.039) together with physical hypoxia; where extracellular BCECF varied from 4.64 ± 0.29 μg/ml to 8.91 ± 0.8 μg/ml (p = 0.034). In parallel, we observed no significant difference between the two exposures. The relative importance between Pgp and MRP-1 was evaluated with two specific inhibitors of Pgp and MRP-1, *i*.*e*., verapamil and probenecid, respectively. We observed no significant difference between the two transporters.

In a second time, we studied the expression of Pgp and MRP-1 proteins in our *in vitro* BBB model after hydralazine or physical hypoxia exposure (2h) in order to understand the increase in the pump’s observed activity (Fig 7C). MRP-1 expression increased after incubation each exposure. For physical hypoxia MRP-1 increased by 27% and by 18% for hydralazine. No significant difference between control and hypoxia exposures was observed. Pgp expression significantly increased by 41% (p = 0.015) and 55% (p = 0.016) for physical hypoxia and hydralazine, respectively. There was no significant difference between the two hypoxia exposures (p = 0.065).

This study on transendothelial transport mediated efflux transporters showed that the functionality of Pgp and MRP-1 was more active on the apical membrane and this functionality was increased after hypoxic stress. This increase was explained by an overexpression of Pgp and MRP-1 proteins.

## Discussion

Some neurological diseases are associated with BBB disruption. The BBB is an essential structure of the neurovascular unit because it protects the brain from ionic variations and regulates the exchange of molecules between blood circulation and brain tissue. These functions are mainly ensured by TJ proteins and efflux transporters[6,27]. These TJ proteins create a physical dam to paracellular diffusion; whereas efflux transporters act as a barrier system since they actively pump compounds out of cerebral microvascular endothelial cells, which reduce the exposure of the central nervous system to drugs. Hypoxia seems to be a characteristic of these neurological diseases and also a major stress factor that can induce BBB disruption. The cellular response to hypoxia is driven by the HIF-1 pathway [12]. However, elucidation of cellular or molecular mechanisms induced by hypoxic stress, is difficult with physical hypoxia since HIF-1α has a short half-life. In this regard, we present in this study an original method of hypoxic stress induction via a mimetic agent of the hypoxia pathway. We decided to validate our induction method by comparing the capacity of a new potential mimetic agent, hydralazine, to physical hypoxia (2% O_2_) in order to create a hypoxic state. This original induction method was set up to understand cellular mechanisms involved by hypoxic stress on an *in vitro* BBB model. Our *in vitro* BBB model was composed of the immortalized cell line bEnd.3 and the immortalized C6 cell line. The objective of our study was to establish a model and methodology of hypoxic induction, which requires a large number of cells and a strong reproducibility rate. These points are not always provided by the primary culture, even if primary cultures are more physiological than immortalized cell lines to set up a BBB model. This is why immortalized cell lines are good alternative methods.

In a first step, we demonstrated that these two exposures (hydralazine or physical hypoxia) were able to induce a hypoxic state on our cells since we observed an increase of HIF-1α protein expression. We found that hydralazine induced an overexpression of HIF-1α after 2 h at 100 μM (Fig 3). This has been also confirmed by other researchers using this agent [18] or by physical hypoxia (Fig 3). We decided to use this concentration to investigate the consequences of hypoxic stress on our *in vitro* BBB model. Our results showed that hypoxic stress induced by hydralazine or physical hypoxia significantly decreased TEER value (Fig 4) and significantly increased membrane permeability to fluorescein sodium (Fig 5). Taken together, these results demonstrated that BBB lost its physical barrier properties after hypoxic stress since ionic compounds (measured by TEER value) and molecules (measured by fluorescein sodium) could cross cell membranes. To understand this loss, we investigated the expression of TJ proteins since they are in charge of these physical barrier properties. Hypoxic stress significantly decreased ZO-1 but not occludin expression (Fig 6). This result confirmed those obtained by Engelhard et al with a CoCl_2_ approach (another mimetic agent of the hypoxia pathway)[12]. This suggested that HIF-1α signalling impacts ZO-1 localization but has no effect on occludin localization. This must be explained by the fact that ZO-1 acts an important role in the development and barrier maintenance of the BBB[28]. Moreover, animal experiments (knockdown for occludin) showed that occludin was not essential for the establishment of TJ complexes despite the fact a loss of this protein was involved in many diseases. Occludin would participate in the regulation of the BBB and more particularly in the regulation of calcium flux through the BBB[29]. Then we investigated the impact of hypoxic stress on efflux transporters as they confer a protection for the brain by rejecting potential dangerous compounds. We showed that Pgp and MRP-1 were more present on apical membranes of brain endothelial cells (Fig 7A), which confirmed literature data[30–32]. We further investigated the impact of hypoxic stress on this apical transport (Fig 7B). Indeed, we showed that endothelial cells of our *in vitro* BBB model released more BCECF, with hydralazine or physical hypoxia, than cells exposed to normoxia. This increase demonstrated that Pgp and MRP-1 were significantly active since they rejected more BCECF during hypoxic stress; but we noticed no significant difference between the two pumps. This result was shown in tumours cells which are rather hypoxic cells. In these cells, HIF-1α was overexpressed and was associated with an increase of Pgp and MRP-1 activity, which explained a chemoresistance for these cells[33]. Finally, we showed that this increase in activity was linked to an overexpression of Pgp and MRP-1 protein after hypoxic stress (Fig 7C). Taken together, these results demonstrated that cells established a defence mechanism to protect the brain against hypoxic stress; but it would create a resistance to therapy, as it has been described for anti-epileptic drugs and chemotherapy[34]. This would be taken into consideration during the development of therapeutics.

This study was undertaken to validate hydralazine as a suitable hypoxia inductor in order to investigate and understand cellular and molecular impacts of hypoxic stress on the BBB. Our results presented hydralazine as a suitable candidate to create a hypoxic state in our in vitro BBB model, since it was able to induce HIF-1α as physical hypoxia and with few cytotoxicity effects. This result is in line with those described in the literature [18]. Hypoxic stress induced by hydralazine led to an increase of permeability due to a loss of ZO-1 at TJ sites. In contrast, this hypoxic stress induced a defence cellular mechanism with an overexpression of Pgp and MRP-1 which associated with an increase of the pump’s activity. Finally, we showed similar results to those obtained by physical hypoxia (2% of O_2_ during 2 h). However, hypoxic alternative methods with mimetic agents could limit studies. Indeed, Shweta et al, showed that cobalt chloride, another mimetic agent, induced an inflammatory response by producing proinflammatory molecules like TNF-α, IL-6 or NO[35]. Moreover, Zhigalova et al, explained that cobalt chloride could activate other molecular pathways[36]. These activations of molecular pathways have not been shown with hydralazine in the literature because of the few studies on this molecule[18], but it could be a possibility. Nevertheless, hydralazine seems to be a good way to understand the first cellular and molecular steps caused by hypoxia on BBB, since our results with hydralazine showed similar results to those obtained with physical hypoxia.

## Conclusion

Induction of hypoxic stress using hydralazine is an innovative and acceptable method to understand the early stages of the consequences of hypoxic stress on the BBB. This induction method has many advantages because hypoxic stress is controlled, reproducible and reversible. It is also less expensive. We are confident that this innovative induction method will allow the understanding of cellular and molecular mechanisms activated at the BBB during pathological states and will bring us some necessary answers for the development of therapeutics.

## Supporting Information

S1 Fig Cytotoxicity effect of drugs in bEnd.3 cells. Cells were incubated with 100 μM of hydralazine during 2 h and 24 h. Cytotoxicity was measured by the LDH release method. The results are presented as mean value for triplicate.
(TIF)

## References

[1] Hawkins BT, Davis TP. The blood-brain barrier/neurovascular unit in health and disease. Pharmacol Rev. 2005;57: 173–185. 10.1124/pr.57.2.4 15914466

[2] Helms HC, Abbott NJ, Burek M, Cecchelli R, Couraud P-O, Deli MA, et al In vitro models of the blood-brain barrier: An overview of commonly used brain endothelial cell culture models and guidelines for their use. J Cereb Blood Flow Metab Off J Int Soc Cereb Blood Flow Metab. 2016; 10.1177/0271678X16630991 PMC4853841 26868179

[3] Cioni C, Turlizzi E, Zanelli U, Oliveri G, Annunziata P. Expression of Tight Junction and Drug Efflux Transporter Proteins in an in vitro Model of Human Blood-Brain Barrier. Front Psychiatry. 2012;3: 47 10.3389/fpsyt.2012.00047 22593745 PMC3350029

[4] Brown RC, Morris AP, O’Neil RG. Tight junction protein expression and barrier properties of immortalized mouse brain microvessel endothelial cells. Brain Res. 2007;1130: 17–30. 10.1016/j.brainres.2006.10.083 17169347 PMC1995120

[5] Kido Y, Tamai I, Nakanishi T, Kagami T, Hirosawa I, Sai Y, et al Evaluation of blood-brain barrier transporters by co-culture of brain capillary endothelial cells with astrocytes. Drug Metab Pharmacokinet. 2002;17: 34–41. 15618650 10.2133/dmpk.17.34

[6] Al Ahmad A, Taboada CB, Gassmann M, Ogunshola OO. Astrocytes and pericytes differentially modulate blood-brain barrier characteristics during development and hypoxic insult. J Cereb Blood Flow Metab Off J Int Soc Cereb Blood Flow Metab. 2011;31: 693–705. 10.1038/jcbfm.2010.148 PMC3049523 20827262

[7] Dehouck MP, Méresse S, Delorme P, Fruchart JC, Cecchelli R. An easier, reproducible, and mass-production method to study the blood-brain barrier in vitro. J Neurochem. 1990;54: 1798–1801. 2182777 10.1111/j.1471-4159.1990.tb01236.x

[8] Cecchelli R, Aday S, Sevin E, Almeida C, Culot M, Dehouck L, et al A stable and reproducible human blood-brain barrier model derived from hematopoietic stem cells. PloS One. 2014;9: e99733 10.1371/journal.pone.0099733 24936790 PMC4061029

[9] Fischer S, Wobben M, Kleinstück J, Renz D, Schaper W. Effect of astroglial cells on hypoxia-induced permeability in PBMEC cells. Am J Physiol Cell Physiol. 2000;279: C935–944. 11003573 10.1152/ajpcell.2000.279.4.C935

[10] Sandoval KE, Witt KA. Blood-brain barrier tight junction permeability and ischemic stroke. Neurobiol Dis. 2008;32: 200–219. 10.1016/j.nbd.2008.08.005 18790057

[11] Al Ahmad A, Gassmann M, Ogunshola OO. Involvement of oxidative stress in hypoxia-induced blood-brain barrier breakdown. Microvasc Res. 2012;84: 222–225. 10.1016/j.mvr.2012.05.008 22668821

[12] Engelhardt S, Al-Ahmad AJ, Gassmann M, Ogunshola OO. Hypoxia selectively disrupts brain microvascular endothelial tight junction complexes through a hypoxia-inducible factor-1 (HIF-1) dependent mechanism. J Cell Physiol. 2014;229: 1096–1105. 10.1002/jcp.24544 24375098

[13] Ogunshola OO, Al-Ahmad A. HIF-1 at the blood-brain barrier: a mediator of permeability? High Alt Med Biol. 2012;13: 153–161. 10.1089/ham.2012.1052 22994514

[14] Yang S-J, Pyen J, Lee I, Lee H, Kim Y, Kim T. Cobalt chloride-induced apoptosis and extracellular signal-regulated protein kinase 1/2 activation in rat C6 glioma cells. J Biochem Mol Biol. 2004;37: 480–486. 15469737 10.5483/bmbrep.2004.37.4.480

[15] Wang Y, Tang Z, Xue R, Singh GK, Liu W, Lv Y, et al Differential response to CoCl2-stimulated hypoxia on HIF-1α, VEGF, and MMP-2 expression in ligament cells. Mol Cell Biochem. 2012;360: 235–242. 10.1007/s11010-011-1061-5 21938405

[16] Cervellati F, Cervellati C, Romani A, Cremonini E, Sticozzi C, Belmonte G, et al Hypoxia induces cell damage via oxidative stress in retinal epithelial cells. Free Radic Res. 2014;48: 303–312. 10.3109/10715762.2013.867484 24286355

[17] Rodrigues SF, de Oliveira MA, dos Santos RA, Soares AG, de Cássia Tostes R, Carvalho MHC, et al Hydralazine reduces leukocyte migration through different mechanisms in spontaneously hypertensive and normotensive rats. Eur J Pharmacol. 2008;589: 206–214. 10.1016/j.ejphar.2008.05.003 18554582

[18] Knowles HJ, Tian Y-M, Mole DR, Harris AL. Novel mechanism of action for hydralazine: induction of hypoxia-inducible factor-1alpha, vascular endothelial growth factor, and angiogenesis by inhibition of prolyl hydroxylases. Circ Res. 2004;95: 162–169. 10.1161/01.RES.0000134924.89412.70 15192023

[19] Horsman MR, Nordsmark M, Høyer M, Overgaard J. Direct evidence that hydralazine can induce hypoxia in both transplanted and spontaneous murine tumours. Br J Cancer. 1995;72: 1474–1478. 8519662 10.1038/bjc.1995.532 PMC2034065

[20] Wuest DM, Wing AM, Lee KH. Membrane configuration optimization for a murine in vitro blood-brain barrier model. J Neurosci Methods. 2013;212: 211–221. 10.1016/j.jneumeth.2012.10.016 23131353

[21] Hayashi K, Nakao S, Nakaoke R, Nakagawa S, Kitagawa N, Niwa M. Effects of hypoxia on endothelial/pericytic co-culture model of the blood-brain barrier. Regul Pept. 2004;123: 77–83. 10.1016/j.regpep.2004.05.023 15518896

[22] Eigenmann DE, Xue G, Kim KS, Moses AV, Hamburger M, Oufir M. Comparative study of four immortalized human brain capillary endothelial cell lines, hCMEC/D3, hBMEC, TY10, and BB19, and optimization of culture conditions, for an in vitro blood-brain barrier model for drug permeability studies. Fluids Barriers CNS. 2013;10: 33 10.1186/2045-8118-10-33 24262108 PMC4176484

[23] Brown RC, Mark KS, Egleton RD, Huber JD, Burroughs AR, Davis TP. Protection against hypoxia-induced increase in blood-brain barrier permeability: role of tight junction proteins and NFkappaB. J Cell Sci. 2003;116: 693–700. 12538770 10.1242/jcs.00264

[24] Deli MA, Abrahám CS, Kataoka Y, Niwa M. Permeability studies on in vitro blood-brain barrier models: physiology, pathology, and pharmacology. Cell Mol Neurobiol. 2005;25: 59–127. 15962509 10.1007/s10571-004-1377-8 PMC11529645

[25] Perek N, Le Jeune N, Denoyer D, Dubois F. MRP-1 protein expression and glutathione content of in vitro tumor cell lines derived from human glioma carcinoma U-87-MG do not interact with 99mTc-glucarate uptake. Cancer Biother Radiopharm. 2005;20: 391–400. 10.1089/cbr.2005.20.391 16114987

[26] Perek N, Denoyer D, Dubois F, Koumanov F. Malignant gliomas display altered plasma membrane potential and pH regulation—interaction with Tc-99m-MIBI and Tc-99m-Tetrofosmin uptakes. Gen Physiol Biophys. 2002;21: 381–404. 12693711

[27] Berezowski V, Landry C, Dehouck M-P, Cecchelli R, Fenart L. Contribution of glial cells and pericytes to the mRNA profiles of P-glycoprotein and multidrug resistance-associated proteins in an in vitro model of the blood-brain barrier. Brain Res. 2004;1018: 1–9. 10.1016/j.brainres.2004.05.092 15262198

[28] Katsuno T, Umeda K, Matsui T, Hata M, Tamura A, Itoh M, et al Deficiency of zonula occludens-1 causes embryonic lethal phenotype associated with defected yolk sac angiogenesis and apoptosis of embryonic cells. Mol Biol Cell. 2008;19: 2465–2475. 10.1091/mbc.E07-12-1215 18353970 PMC2397322

[29] Saitou M, Furuse M, Sasaki H, Schulzke JD, Fromm M, Takano H, et al Complex phenotype of mice lacking occludin, a component of tight junction strands. Mol Biol Cell. 2000;11: 4131–4142. 11102513 10.1091/mbc.11.12.4131 PMC15062

[30] Fang W, Lv P, Geng X, Shang E, Yang Q, Sha L, et al Penetration of verapamil across blood brain barrier following cerebral ischemia depending on both paracellular pathway and P-glycoprotein transportation. Neurochem Int. 2013;62: 23–30. 10.1016/j.neuint.2012.10.012 23142723

[31] Felix RA, Barrand MA. P-glycoprotein expression in rat brain endothelial cells: evidence for regulation by transient oxidative stress. J Neurochem. 2002;80: 64–72. 11796744 10.1046/j.0022-3042.2001.00660.x

[32] Nies AT, Jedlitschky G, König J, Herold-Mende C, Steiner HH, Schmitt H-P, et al Expression and immunolocalization of the multidrug resistance proteins, MRP1-MRP6 (ABCC1-ABCC6), in human brain. Neuroscience. 2004;129: 349–360. 10.1016/j.neuroscience.2004.07.051 15501592

[33] Chen Z-S, Lee K, Walther S, Raftogianis RB, Kuwano M, Zeng H, et al Analysis of methotrexate and folate transport by multidrug resistance protein 4 (ABCC4): MRP4 is a component of the methotrexate efflux system. Cancer Res. 2002;62: 3144–3150. 12036927

[34] Xiao-Dong L, Zhi-Hong Y, Hui-Wen Y. Repetitive/temporal hypoxia increased P-glycoprotein expression in cultured rat brain microvascular endothelial cells in vitro. Neurosci Lett. 2008;432: 184–187. 10.1016/j.neulet.2007.12.017 18241990

[35] Shweta, Mishra KP, Chanda S, Singh SB, Ganju L. A comparative immunological analysis of CoCl2 treated cells with in vitro hypoxic exposure. Biometals Int J Role Met Ions Biol Biochem Med. 2015;28: 175–185. 10.1007/s10534-014-9813-9 25511110

[36] Zhigalova N, Artemov A, Mazur A, Prokhortchouk E. Transcriptome sequencing revealed differences in the response of renal cancer cells to hypoxia and CoCl 2 treatment. F1000Research. 2015;4: 1518 10.12688/f1000research.7571.1 26925226 PMC4712771

