# Supplementary figures and images for: Hypoxic Stress Induced by Hydralazine Leads to a Loss of Blood-Brain Barrier Integrity and an Increase in Efflux Transporter Activity
Source: PLoS One. 2016 Jun 23;11(6):e0158010. doi: 10.1371/journal.pone.0158010 (PMC4919080; doi:10.1371/journal.pone.0158010)

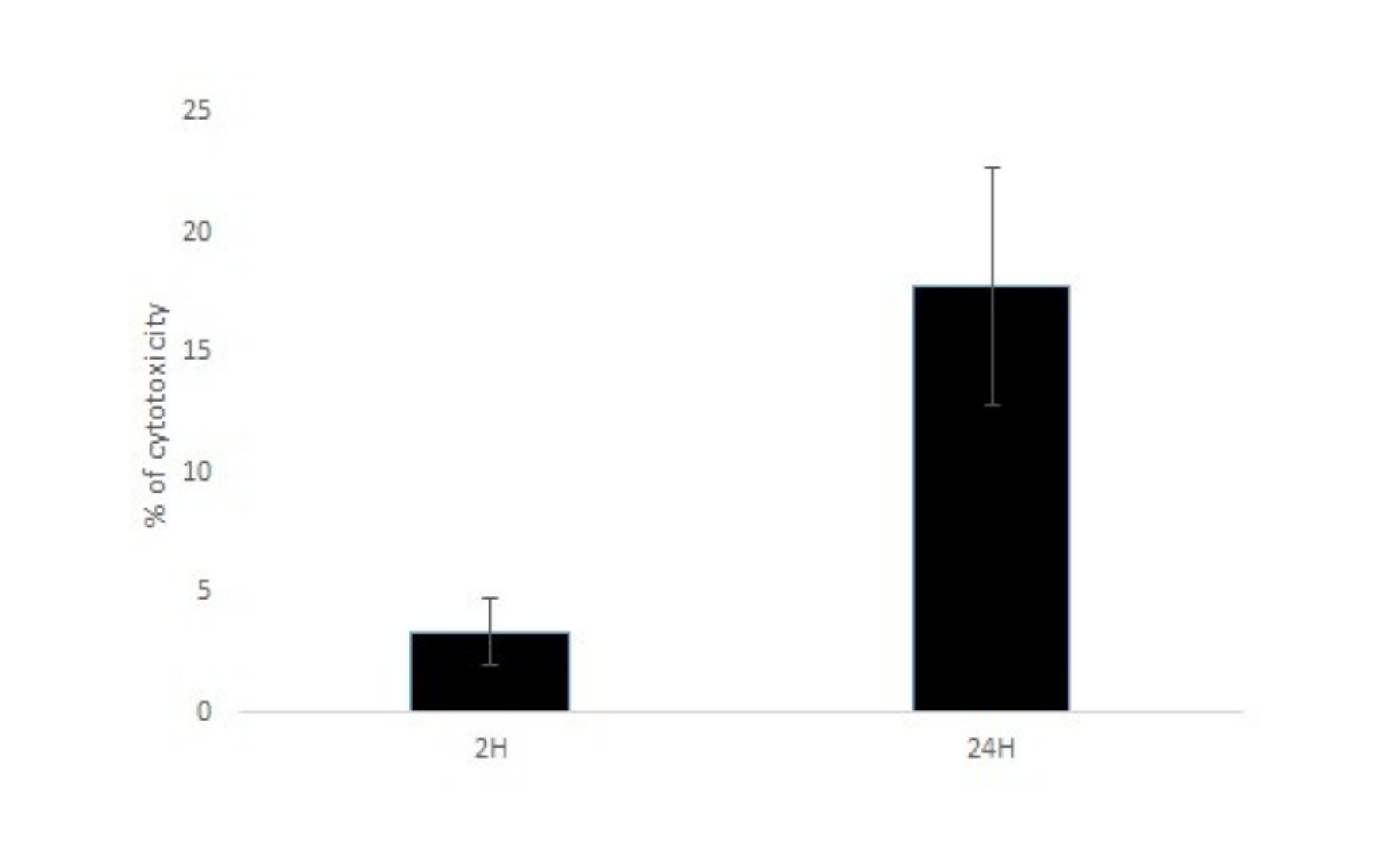

Supplement: S1 Fig — Cells were incubated with 100 μM of hydralazine during 2 h and 24 h. Cytotoxicity was measured by the LDH release method. The results are presented as mean value for triplicate. (TIF) [file pone.0158010.s001.tif]
